# Association between increasing agricultural use of 2,4-D and population biomarkers of exposure: findings from the National Health and Nutrition Examination Survey, 2001–2014

**DOI:** 10.1186/s12940-021-00815-x

**Published:** 2022-02-10

**Authors:** Marlaina S. Freisthler, C. Rebecca Robbins, Charles M. Benbrook, Heather A. Young, David M. Haas, Paul D. Winchester, Melissa J. Perry

**Affiliations:** 1 Department of Environmental and Occupational Health, Milken Institute School of Public Health, The George Washington University, 950 New Hampshire Ave NW Suite 400, Washington, DC 20052 USA; 2 Heartland Health Research Alliance, Ltd., Brookfield, WI USA; 3 Department of Obstetrics and Gynecology, Indiana University School of Medicine, Indianapolis, Indiana USA; 4 Neonatology, Indiana University School of Medicine/Riley Hospital, Indianapolis, Indiana USA

**Keywords:** Environmental epidemiology, Environmental exposure, Pesticides

## Abstract

**Background:**

2,4-Dichlorophenoxyacetic acid (2,4-D) is one of the most extensively used herbicides in the United States. In 2012, 2,4-D was the most widely used herbicide in non-agricultural settings and the fifth most heavily applied pesticide in the US agricultural sector. The objective of this study was to examine trends in 2,4-D urinary biomarker concentrations to determine whether increases in 2,4-D application in agriculture are associated with increases in biomonitoring levels of urine 2,4-D.

**Methods:**

Data from the National Health and Nutrition Examination Survey (NHANES) with available urine 2,4-D biomarker measurements from survey cycles between 2001 and 2014 were utilized. Urine 2,4-D values were dichotomized using the highest limit of detection (LOD) across all cycles (0.40 μg/L or 0.4 ppb). Agricultural use of 2,4-D was estimated by compiling publicly available federal and private pesticide application data. Logistic regression models adjusted for confounders were fitted to evaluate the association between agricultural use of 2,4-D and urine 2,4-D level above the dichotomization threshold.

**Results:**

Of the 14,395 participants included in the study, 4681 (32.5%) had urine 2,4-D levels above the dichotomization threshold. The frequency of participants with high 2,4-D levels increased significantly (*p* < .0001), from a low of 17.1% in 2001–2002 to a high of 39.6% in 2011–2012. The adjusted odds of high urinary 2,4-D concentrations associated with 2,4-D agricultural use (per ten million pounds applied) was 2.268 (95% CI: 1.709, 3.009). Children ages 6–11 years (*n* = 2288) had 2.1 times higher odds of having high 2,4-D urinary concentrations compared to participants aged 20–59 years. Women of childbearing age (age 20–44 years) (*n* = 2172) had 1.85 times higher odds than men of the same age.

**Conclusions:**

Agricultural use of 2,4-D has increased substantially from a low point in 2002 and it is predicted to increase further in the coming decade. Because increasing use is likely to increase population level exposures, the associations seen here between 2,4-D crop application and biomonitoring levels require focused biomonitoring and epidemiological evaluation to determine the extent to which rising use and exposures cause adverse health outcomes among vulnerable populations (particularly children and women of childbearing age) and highly exposed individuals (farmers, other herbicide applicators, and their families).

**Supplementary Information:**

The online version contains supplementary material available at 10.1186/s12940-021-00815-x.

## Introduction

2,4-Dichlorophenoxyacetic acid (2,4-D) is one of the most extensively used herbicides in the United States. Roughly 600 US agricultural and residential use products contain 2,4-D as the active ingredient [1, 2]. In 2012, 2,4-D was the most widely used herbicide in home and garden settings, roughly equal to glyphosate in use in the combined non-agricultural settings, and the fifth most heavily applied pesticide in the US agricultural sector [3].

2,4-D is rapidly absorbed via oral and inhalation routes [4]. In prior evaluations in the Agricultural Health Study (AHS), 71% of 2,4-D applicators from Minnesota and South Carolina had 2,4-D in their urine prior to applying it to their crops, while 100% had it in their urine post-application [5]. AHS research has also reported that 41% of spouses and 62% of children of the agricultural workers had detectable levels of 2,4-D in their urine pre-application [6]. Farm family members are typically exposed to pesticides via multiple routes, including food, drinking water, inhalation and dermal exposure following spray drift or movement of volatile compounds [7]. Those who live near areas of heavy agricultural 2,4-D use often have increased exposure from dermal contact, inhalation of soil particles, and contact with people, clothing, or pets that have been exposed [1].

Non-occupational exposure studies have reported that farm families are not the only population at risk of 2,4-D exposure. Exposure to 2,4-D also occurs via food, water, dust, dirt, and pet exposure in non-agricultural households. An evaluation of exposure in children and their adult caregivers outside of the agricultural context found that 2,4-D was detected in 83 and 98% of household carpet dust samples in six counties each in North Carolina and Ohio, respectively, as well as in more than 85% of participants’ spot urine samples in both locations [8]. The general population is exposed to 2,4-D through ingestion of food and water containing residues of 2,4-D [1], as well as through ambient non-occupational residential exposures, particularly in areas where 2,4-D has been widely used in controlling weeds in lawns and urban spaces [9]. The toxicological profile for 2,4-D indicates that it is highly mobile in soil and has the potential to migrate into groundwater [1], a particular concern for households near high-application areas.

US agricultural application patterns of 2,4-D use over time are showing major increases, particularly since the shift towards weed management through reliance on genetically modified herbicide resistant seeds in the early 2000s triggered the emergence and spread of glyphosate-resistant weeds [2, 10, 11]; however, how these increases are affecting human exposure is not known. As increasing rates of exposure are anticipated due to the planting of herbicide-resistant seeds in conjunction with the wider, more frequent use of multi-ingredient herbicide premixes, innovative biomarker analyses are becoming increasingly essential in evaluating human health impacts. The objective of this study was to examine trends in 2,4-D urinary biomarker concentrations in a nationally representative US population from 2001 to 2014 to determine whether increases in 2,4-D application in agriculture are associated with increases in biomonitoring levels of urine 2,4-D as a measure of population exposure, and to identify any population subgroups who are more likely to be exposed.

## Methods

### Biomonitoring data

The National Health and Nutrition Examination Survey (NHANES) is a continuous, nationally representative, cross-sectional survey of the non-institutionalized civilian US population. It is conducted by the U.S. Centers for Disease Control and Prevention (CDC) and published in two-year waves. Urine 2,4-D has been measured as part of a randomly selected environmental exposure subsample, typically representing around one-third of the overall sample [12]. Spot urine samples collected during scheduled appointments at a mobile examination center were immediately aliquoted and stored at − 20 degrees Celsius prior to shipping for analysis at CDC’s National Center for Environmental Health in Atlanta, Georgia [13].

Although inhalation and dermal routes are important exposure sources in occupational settings, non-occupational 2,4-D exposures in humans are presumed to happen primarily through ingestion [14, 15]. Most of the 2,4-D consumed orally is absorbed in the gastrointestinal tract [15]. Because it is minimally metabolized prior to urinary excretion [1] and retains its parent form in urine, no other associated analytes/metabolites are analyzed to evaluate exposure. An automated solid phase extraction system was used to extract and concentrate 2,4-D from the urine matrix. High-performance liquid chromatography with gradient elution was used to selectively separate 2,4-D, which was then detected, identified, and quantified by a triple quadrupole mass spectrometer with a heated electrospray ionization source [16].

All 2,4-D data files were downloaded from the NHANES website in June 2020. All survey cycles in which 2,4-D biomonitoring data were available except the 1999–2000 cycle were utilized for this analysis. The 1999–2000 cycle was excluded because the 2,4-D limit of detection (LOD) was more than two times higher than the LOD of subsequent surveys (1.0 μg/L or 1 ppb). Because the 2005–2006 surveys and all surveys after the 2013–2014 cycle did not include 2,4-D biomarkers, they were not included in this analysis. The survey cycles utilized were the 2001–02, 2003–04, 2007–08, 2009–10, 2011–12, and 2013–14 cycles. Across these six cycles, a total of 61,778 participants were surveyed with 16,553 participants in the environmental exposure subsample. Urine 2,4-D measurements were available from 15,761 (95.2%) participants. Participants were excluded who had data missing on household income (*n* = 1199), educational background (*n* = 20), status as an agricultural worker (*n* = 22), or spot urine creatinine level (*n* = 8), resulting in a total of 14,395 (90.3%) participants included in this study.

Limits of detection (LOD) for urine 2,4-D metabolites varied by cycle from 0.15–0.40 μg/L, with 5907 samples (41.04%) falling below the respective LOD. Due to the variability of the LODs and the high percentage of results falling below the LOD, urine 2,4-D values were dichotomized using the highest LOD level across all cycles (0.40 μg/L or 0.4 ppb). Approximately one third (32.52%) of detections fell above the dichotomization threshold and were considered the high exposure group, while the remaining two thirds falling below the dichotomization threshold were considered the low/no exposure group. High compared to low urinary concentrations was the main outcome of interest because, while low exposures may pose health risks, higher 2,4-D exposures have been associated with negative health outcomes in prior studies.

### Covariates

In the NHANES survey, participants were asked about the industry in which they were employed and their occupation. Both were coded according to the US Census Bureau’s Industry and Occupation Codes [17]. For this study, a participant was considered an agricultural worker if they reported agricultural sector for either their industry or occupation. Children under age 16 were presumed not to be agricultural workers because they do not participate in the NHANES Occupation Questionnaire.

Adults were considered to be smokers if their cotinine levels on laboratory analysis were greater than 10 ng/mL, or if they self-reported as smokers [18]. They were considered non-smokers if their cotinine levels were less than or equal to 10 ng/mL. If a laboratory value was not available, participants were considered to be non-smokers if they self-reported as a non-smoker. Children under age 12 were not questioned regarding their smoking status and were considered to be non-smokers unless their cotinine value was greater than 10 ng/mL on laboratory analysis [18].

### Pesticide use data

**Fig. 1:**
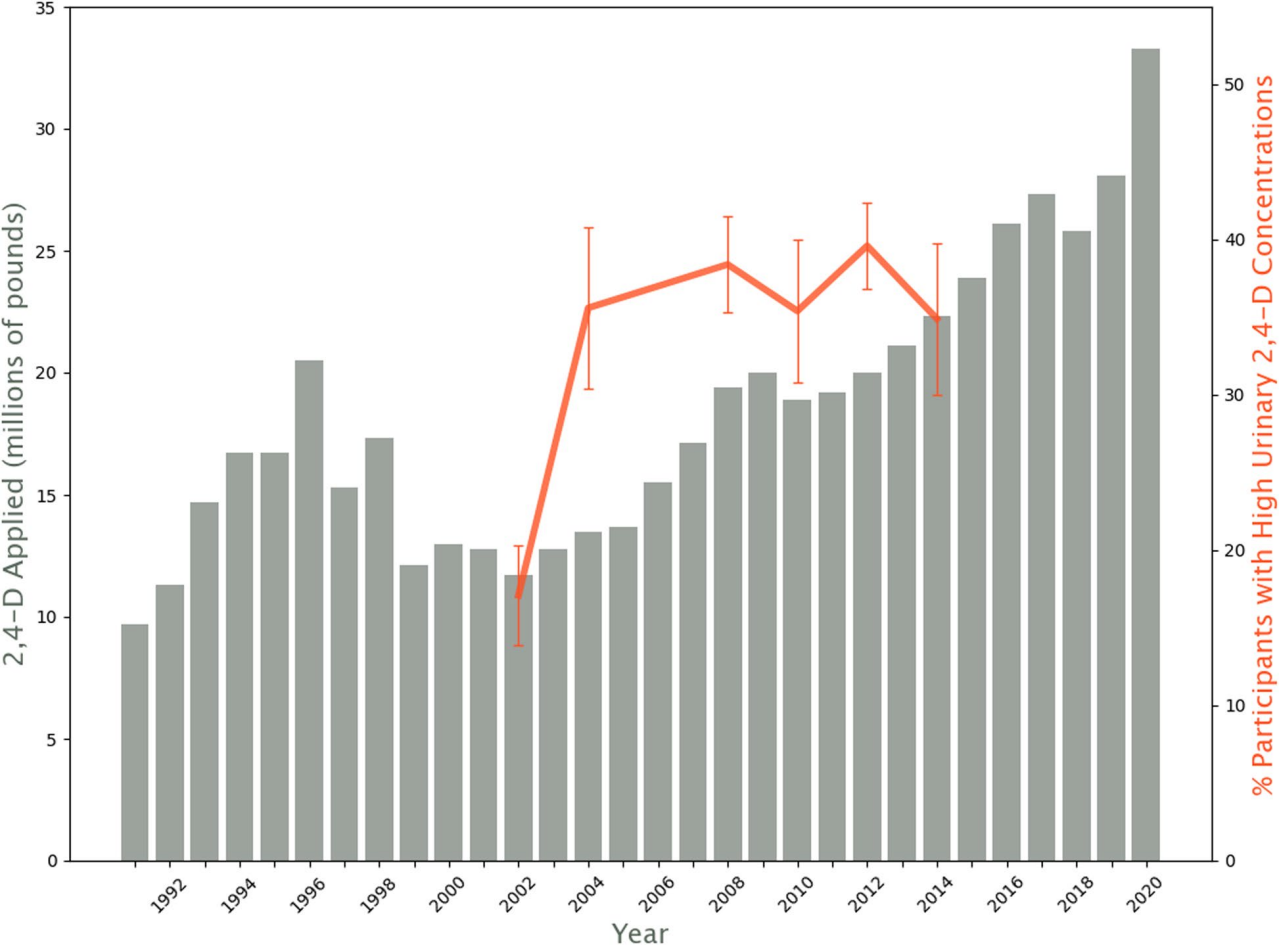
2,4-D Agricultural Use and NHANES Urinary Concentrations Over Time. Legend: Estimated agricultural use of 2,4-D per year in millions-of-pounds (left y-axis bar chart) and percent of NHANES participants with high (> 0.4μg/L urine (or 0.4 ppb) 2,4-D urinary concentrations (right y-axis line chart, with 95% confidence interval bars) by concluding year of NHANES survey

The amount of 2,4-D applied annually was estimated for specific crops using the United States Department of Agriculture (USDA) National Agricultural Statistics Service (NASS) survey data (see Fig. 1 and Additional file 1) using methods described previously [19]. Briefly, since 1990, NASS has reported annually pesticide applications at the national and state level for most major field crops. Fruit crops are surveyed in even years and vegetable crops in odd years. Although the NASS surveys a limited number of crops in any given year and no crop every year, use data is commonly interpolated for years in which data are not available based on the assumption of equal percentage changes year-to-year between two years with reported values. Additional but less granular pesticide use data are available in periodic U.S. Environmental Protection Agency (EPA) reports. These reports provide the most complete estimates of total use in both agriculture and non-agricultural settings; the most recent report in this EPA series provides data through 2012 [3]. Detailed proprietary data are issued annually by private companies and are used by the EPA to augment the results from annual USDA surveys. Another dataset is compiled and shared publicly by the United States Geological Service.

Results from NASS and EPA were compiled in the Pesticide Use Data System (PUDS) [19] to produce annual data from 1974 through 2020 on 2,4-D use in agriculture. Annual USDA surveys report use data for multiple forms of 2,4-D. In PUDS, conglomerate values for a given pesticide integrate multiple forms of an active ingredients into a single set of data (see Additional file 2). To examine exposure biomarkers in relation to per-year pesticide use, the average amount of aggregate 2,4-D applied per year within an NHANES cycle was determined by summing the amount of 2,4-D applied in each of the two years and then dividing by two.

### Statistical analysis

NHANES oversamples some minority demographics (e.g., race/ethnicity and older age) in order to better identify health trends and then applies sample weights to ensure that the sample is nationally representative [20]. It also uses clustering in its sampling strategy, to account for non-random community and family relationships among survey participants [20]. To account for these factors, analyses were performed using survey procedures to adjust for nonrandom sampling design and sample population weights [20].

Sample weights and degrees of freedom were calculated according to the NHANES analytic guidelines [21]. Separate sampling weights are provided for each NHANES cycle. Because each sample weight represents only one cycle, and six cycles were analyzed, sampling weights for this analysis were calculated by assigning a value of one-sixth of the two-year subsample weights, in accordance with recommended procedures [21]. Degrees of freedom were calculated as the number of clusters in the second level of sampling (primary sampling units) minus the number of clusters in the first sampling unit (strata) [21]. With 94 degrees of freedom, a critical value of + 1.986 was derived from the t-distribution for the calculation of confidence intervals in all inferential analyses. Taylor series linearization was used to compute variance estimates [22]. Analyses were conducted in SAS version 9.4 (SAS Institute, Cary, NC).

Multivariable logistic regression models were used to assess the relationship between urinary 2,4-D concentrations and the average amount of 2,4-D used in crop applications per year across the survey cycle (in ten-million-pound units), adjusting for potential covariates. Based on a priori assumptions from prior research on pesticide biomarkers, age, sex (male versus female), family poverty to income ratio, race (non-Hispanic Black, Mexican American, and Other Race/Ethnicity versus non-Hispanic White), and agricultural worker status (yes/no) were included as covariates. All regression models controlled for creatinine level to account for urinary dilution, which can affect accuracy in urinary pesticide detections [23]. Smoking status, education level (Grade 8 or less, Grades 9–12, and high school graduate versus some college) and season of testing (May 1–October 31 vs. November 1–April 30) were evaluated as potential confounders and were retained if their inclusion significantly changed (> 10%) the odds ratio of the main association in any model. Additional multivariable analyses for target age group and sex were performed using similar modelling techniques to evaluate associations in high-risk subgroups. These subgroups were identified a priori because of interest in exposure levels relative to developmental and reproductive vulnerability [24]. Specifically, models were fitted for age groups 6–11 years, 12–19 years, 20–59 years, and 60+ years, and separately for men and women of childbearing age (20–44 years). The same covariates were initially evaluated for all models. A two-sided *p*-value < 0.05 was considered statistically significant.

## Results

Descriptive statistics for the total sample are detailed in Table 1. Of the 14,395 participants included in the study, 4681 (32.5%) had urine 2,4-D levels above 0.40 μg/L or 0.4 ppb. Table 1 contains unadjusted odds ratios of higher urinary 2,4-D concentration by predictor variables. Many of the unadjusted demographic analyses showed significant associations with high 2,4-D concentrations including by gender, race, and poverty level. Men had a higher odds of high 2,4-D urinary concentrations compared to women (OR: 1.52, 95% CI: 1.39, 1.67). Non-Hispanic White participants had higher odds of high urinary 2,4-D concentrations compared to Non-Hispanic Black participants (OR: 0.83, 95% CI: 0.72, 0.95), Mexican American participants (OR: 0.86, 95% CI 0.73, 1.01), and participants of other races/ethnicities (OR: 0.70, 95% CI: 0.60, 0.83). Participants with an income over 200% of the poverty level had higher odds compared to participants with income levels 100–200% of poverty level (OR: 0.82, 95% CI: 0.73, 0.91) and up to 100% of the poverty level (OR: 0.79, 95% CI: 0.71, 0.89). Participants who worked in the agricultural sector had higher odds than those who did not (OR: 2.23, 95% CI: 1.33, 3.73), and those who did not smoke had higher odds than those who did (OR: 0.71, 95% CI: 0.63, 0.81). By age, compared to participants aged 20–59, the odds were higher for children aged 6–11 years (OR: 1.60, 95% CI: 1.40, 1.83) and 12–19 years (OR: 1.16, 95% CI: 1.01, 1.33), as well as for participants aged 60 years or older (OR: 1.24, 95% CI: 1.11, 1.37).

**Table 1:** Participant Characteristics and Distribution of Higher Urine 2,4-D Concentrations. Legend: Distribution of high (> 0.4 μg/L urine) 2,4-D urinary concentrations and associations with key variables in unadjusted models. * = reference level

Variable	Frequency (%)	Population Estimate	High Urine 2,4-D in Sample	Estimated Population with High Exposure	% with High 2,4-D (95% CI)	Unadjusted Odds Ratio (95% CI)
Total	14,395	244,607,215	4681	82,642,980	33.8 (32.1, 35.5)	
**Survey Cycle**						
2001–2002	2685 (15.1)	37,004,806	457	6,332,340	17.1 (13.9, 20.3)	ref
2003–2004	2347 (16.4)	40,112,380	855	14,269,430	35.6 (30.4, 40.8)	2.67 (1.99, 3.70)
2007–2008	2311 (16.4)	40,153,846	898	15,427,421	38.4 (35.3, 41.5)	3.02 (2.32, 3.94)
2009–2010	2450 (16.9)	41,228,734	823	14,582,297	35.4 (30.8, 40.0)	2.65 (1.95, 3.61)
2011–2012	2146 (17.3)	42,213,815	791	16,716,965	39.6 (36.8, 42.4)	3.17 (2.45, 4.11)
2013–2014	2456 (17.9)	43,893,633	857	15,314,527	34.9 (30.0, 39.8)	2.60 (1.89, 3.56)
**Sex**						
Male	7099 (49.0)	119,753,832	2614	46,199,838	38.6 (36.5, 40.7)	1.521 (1.39, 1.67)
Female	7296 (51.0)	124,853,382	2067	36,443,142	29.2 (27.3, 31.1)	ref
**Race/Ethnicity**						
Non-Hispanic White	5922 (67.1)	164,017,859	2068	58,251,760	35.5 (33.3, 37.7)	ref
Non-Hispanic Black	3313 (11.7)	28,583,574	1046	601,943	31.3 (28.8, 33.8)	0.83 (0.72, 0.95)
Mexican American	2850 (9.0)	21,996,531	887	7,054,921	32.1 (29.0, 35.2)	0.86 (0.73, 1.01)
Other	2310 (12.3)	30,009,251	680	8,389,887	28.0 (25.1, 30.9)	0.70 (0.60, 0.83)
**Age (years)**						
6–11	2288 (9.2)	22,586,413	874	9,571,185	42.4 (38.8, 46.0)	1.60 (1.40, 1.83)
12–19	2846 (12.1)	29,636,700	914	10,290,245	34.7 (31.5, 37.9)	1.16 (1.01, 1.33)
20–59	6243 (60.0)	146,678,123	1875	46,214,496	31.5 (29.7, 33.3)	ref
60+	3018 (18.7)	45,705,978	1018	16,567,054	36.2 (33.7, 38.7)	1.24 (1.11, 1.37)
**Education**						
Grade 8 or Less	4569 (18.9)	46,298,948	1585	17,680,657	38.2 (35.5, 40.9)	1.34 (1.16, 1.55)
Grade 9–12	2615 (14.4)	35,342,844	799	10,865,543	30.7 (28.4, 33.0)	0.96 (0.84, 1.11)
High School Graduate	2272 (18.3)	44,797,645	676	14,125,909	31.5 (28.5, 34.5)	ref
Any College	4939 (48.3)	118,167,779	1621	39,970,871	33.8 (31.8, 35.8)	1.11 (0.97, 1.27)
**Poverty Income Ratio**						
Up to 100%	3638 (16.6)	40,513,665	1082	12,330,209	30.4 (28.3, 32.5)	0.79 (0.71, 0.89)
100–200%	3898 (21.5)	52,546,663	1228	16,365,202	31.1 (28.8, 33.4)	0.82 (0.73, 0.91)
Over 200%	6859 (62.0)	151,546,887	2371	53,947,570	35.6 (33.6, 37.6)	ref
**Agricultural Worker**						
No	14,256 (98.9)	242,014,960	4620	81,271,238	33.6 (31.9, 35.3)	ref
Yes	139 (1.1)	2,592,255	61	1,371,742	52.9 (46.3, 59.5)	2.23 (1.33, 3.73)
**Smoking Status**						
No	11,444 (76.3)	186,588,955	3869	66,274,113	35.5 (33.7, 37.3)	ref
Yes	2951 (23.7)	58,018,260	812	16,368,867	28.2 (25.6, 30.8)	0.71 (0.63, 0.81)
**Season**						
May 1–October 31	7469 (59.0)	100,371,111	2446	50,441,699	35.0 (32.5, 37.5)	1.14 (0.97, 1.33)
November 1–April 30	6926 (41.0)	144,236,103	2235	32,201,281	32.1 (30.9, 33.3)	ref
**Water Source**						
Water Company	8646 (84.6)	132,271,816	2669	42,110,899	31.8 (29.6, 34.0)	1.04 (0.87, 1.24)
Well	1020 (15.4)	24,127,699	315	7,487,755	31.0 (27.3, 34.7)	ref

As detailed in Fig. 1, the percent of participants with high 2,4-D urinary concentrations increased significantly over the series of surveys (*p* < .0001), from a low of 17.1% in the 2001–2002 survey to a high of 39.6% in the 2011–2012 survey.

Results of bivariate and multivariable logistic regression analyses for all participants and for each age and sex group of interest are reported in Table 2. In the full sample, the unadjusted odds ratio for high urinary 2,4-D concentration associated with pounds of 2,4-D utilized in crop applications (per ten-million-pound unit) was 1.85 (95% CI: 1.44, 2.38, *p* < .0001). In the adjusted models, all covariates selected a priori (age, sex, poverty ratio, race/ethnicity, and agricultural worker status) were retained. Education, smoking, and season of testing were not retained as covariates due to lack of change in effect.

**Table 2:** Adjusted and Unadjusted Associations Between Agricultural Application of 2,4-D and NHANES Urine 2,4-D Concentrations. Legend: Unadjusted and adjusted odds of high 2,4-D urinary concentrations by agricultural use of 2,4-D, age, gender, and other key variables for NHANES participants 2001–2014

Variable	All Participants	Age 6–11	Age 12–20	Age 20–59	Age 60+	Female, Age 20–44	Male, Age 20–44
	***n*** = 14,395	***n*** = 2288	***n*** = 2846	***n*** = 6243	***n*** = 3018	***n*** = 2172	***n*** = 1932
**Unadjusted OR (95% CI)**							
**Agricultural** **2,4-D Applied**^**a**^	1.85 (1.44, 2.38)	3.41 (2.18, 5.34)	1.77 (1.20, 2.60)	2.54 (1.18, 2.01)	2.47 (1.82, 3.36)	2.29 (1.54, 3.40)	1.24 (0.85, 1.80)
**Adjusted OR (95% CI)**							
**Agricultural** **2,4-D Applied**	2.27 (1.71, 3.01)	4.24 (2.52, 7.13)	2.38 (1.54, 3.68)	1.99 (1.45, 2.73)	2.70 (1.87, 3.91)	2.87 (1.82, 4.53)	1.55 (1.02, 2.36)
**Age**	1.00 (1.00, 1.01)	0.87 (0.81, 0.94)	0.93 (0.88, 0.99)	1.02 (1.01, 1.03)	1.02 (1.01, 1.04)	1.01 (0.99–1.04)	1.01 (1.00–1.03)
**Gender**^**b**^	0.87 (0.79, 0.97)	0.94 (0.74, 1.18)	0.78 (0.62, 0.99)	0.94 (0.81, 1.10)	0.88 (0.68, 1.14)	–	–
**Race/Ethnicity**							
Non-Hispanic White	ref	ref	ref	ref	ref	ref	ref
Mexican American	0.83 (0.68, 1.02)	0.68 (0.49, 0.96)	0.87 (0.63, 1.18)	0.90 (0.69, 1.18)	0.75 (0.53, 1.07)	1.00 (0.66–1.51)	0.76 (0.54–1.07)
Non-Hispanic Black	0.57 (0.48, 0.66)	0.47 (0.34, 0.64)	0.68 (0.49, 0.94)	0.52 (0.42, 0.65)	0.58 (0.45, 0.75)	0.53 (0.38–0.75)	0.45 (0.33–0.62)
Other	0.69 (0.57, 0.82)	0.50 (0.35, 0.72)	0.77 (0.56, 1.08)	0.73 (0.56, 0.94)	0.71 (0.49, 1.04)	0.63 (0.43–0.93)	0.69 (0.49–0.98)
**Poverty Income Ratio**	1.06 (1.02, 1.10)	1.12 (1.04, 1.23)	1.06 (0.97, 1.16)	1.06 (1.00, 1.11)	1.09 (1.01, 1.17)	1.08 (0.98–1.18)	1.01 (0.94–1.08)
**Urine Creatinine (mg/dL)**	1.01 (1.01, 1.01)	1.02 (1.01, 1.02)	1.01 (1.01, 1.01)	1.01 (1.01, 1.01)	1.01 (1.01, 1.01)	1.01 (1.01–1.01)	1.01 (1.01–1.01)
**Agricultural Worker**	2.11 (1.33, 3.36)	⎼	1.81 (0.64, 5.13)	2.64 (1.41, 4.94)	2.34 (0.59, 9.25)	2.26 (0.54–9.42)	2.85 (1.42–5.69)

^a^ in 10-million-pound units

^b^ female vs. male

*ref* reference level for comparison

The overall adjusted odds ratio for pounds of 2,4-D applied was 2.27 (95% CI: 1.71, 3.01, p < .0001). In adjusted models, agricultural work was a significant predictor of higher urinary 2,4-D concentrations overall, as well as for adults aged 20–59 and men aged 20–44. Several of the models showed an increased odds of higher exposure in specific age and sex groups. Children ages 6–11 years (*n* = 2288) had 2.1 times higher odds of having higher urinary 2,4-D concentrations compared to participants aged 20–59 years, while participants aged 60 years and older (*n* = 3018) had 1.36 times higher odds. Within the 6–11- and 12–19-years age ranges, age was a significant predictor, indicating that among children within each age group, younger children had higher odds of higher exposure than older children. Women of childbearing age (age 20–44 years) (*n* = 2172) had odds 1.85 times higher than men of the same age.

## Discussion

This study evaluated the relationship between crop application of 2,4-D and urine biomarkers of exposure in a large, nationally representative sample of 14,395 participants. There were significant associations between crop application of 2,4-D and the percent of NHANES participants with high 2,4-D urinary concentrations (adjusted odds ratio 2.27 (95% CI: 1.71, 3.01, *p* < .0001)). This study demonstrates that as average annual use of 2,4-D increased, individuals had increased odds of having higher urinary 2,4-D concentrations.

Overall, the amount of 2,4-D applied in agriculture has risen nearly 67% between 2012 and 2020, and over 240% between 1991 and 2020 (see Additional file 1), a trend that is unlikely to change due to unabated weed resistance. 2,4-D use began in 1945 and dramatically increased over the next two decades. In the late 1960’s, reliance on 2,4-D declined due to the emergence of other selective herbicides such as atrazine, although it remained the herbicide of choice for most wheat growers [2, 10, 11]. Commercial sale of genetically engineered glyphosate-tolerant “Roundup™ Ready” soybeans and cotton began in 1996. Cost-effectiveness and simplicity of use led to rapid and widespread adoption of the Roundup™ system (see Additional files 3 and 4), causing sales of 2,4-D and all other herbicides to decline sharply in these three crops (see Additional files 5 and 6). By the mid 2000s, three hard-to-control weeds had developed resistance to glyphosate in the southeastern US and were spreading rapidly north and west from farm to farm and across state lines. The problem worsened from a few resistant weeds in scattered fields to economically damaging resistant weed populations in many fields by the mid 2010s [25, 26]. The growing diversity and spread of resistant weeds have forced farmers to add additional herbicides, often including 2,4-D, to their weed control programs [25].

In response to increasing herbicide resistance in weeds, the pesticide industry developed DuoEnlist™ technology, which EPA approved in 2017. This technology couples seeds genetically engineered to tolerate 2,4-D, glyphosate, and 4-Hydroxyphenylpyruvate dioxygenase inhibitors (HPPD inhibitors, aka “fop” herbicides) with new formulations of glyphosate and 2,4-D designed to reduce volatility, drift, and off-target crop damage. Reliance on the DuoEnlist™ system has caused the use of 2,4-D to rise sharply in both soybean and cotton production, with further increases highly likely through around 2025 (see Additional files 7 and 8).

The expected trend of increased use of 2,4-D raises concerns about changes in population exposure, particularly for sensitive populations who may be more vulnerable to harmful effects of exposure. The U.S. EPA provides a “chronic reference dose” (chronic RfD) for certain pesticide chemical exposures. This is defined as “an estimate (with uncertainty spanning perhaps an order of magnitude) of a daily exposure to the human population (including sensitive subgroups) that is likely to be without an appreciable risk of deleterious effects during a lifetime” [27]. For 2,4-D, the chronic RfD based on oral consumption is 0.005 mg/kg/day [28]. A biomonitoring equivalent (BE) provides information about how to compare urinary biomonitoring data to the RfD [28]. Toxicokinetic research indicates that the RfD of 0.005 mg/kg/day of 2,4-D is roughly equivalent to a creatinine adjusted urinary concentration of 300 μg/g in the general population [28]. Prior CDC evaluation of NHANES data indicates urinary concentrations of 2,4-D in the general population continued to be orders of magnitude below the BE of the RfD through 2014 [29]. This is the latest survey in which NHANES includes 2,4-D data, and other biomonitoring studies have confirmed these results [30].

The results of this study deserve careful consideration in the context of herbicide exposure given all that is already known about the human health effects of herbicides from animal and epidemiologic studies. While the carcinogenicity of 2,4-D has been intensively studied and long debated [31, 32], new studies have heightened concern. A 2020 longitudinal biomarker study linked 2,4-D with increased systemic markers of oxidative stress [33]. Association between 2,4-D exposures and non-Hodgkin lymphoma (NHL) have been reported, with a recent meta-analysis showing that highly exposed groups experience an elevated relative risk of NHL (RR = 1.73, CI: 1.10–2.72) [34]. The risk of pediatric leukemia is increased in children residing near areas sprayed heavily with herbicides, including 2,4-D and dicamba [35], raising concerns about the impact of exposure to this herbicide on pediatric populations.

Non-cancer outcomes such as birth defects and pediatric anatomical abnormalities have also been linked to 2,4-D. A 1996 study linked licensed pesticide applicators to birth defects from the state birth registry and found an increased rate of birth defects in children of applicators who applied chlorophenoxy herbicides such as 2,4-D [36]. More recently, a case control study assessing birth defects in infants found an association between 2,4-D exposure and hypertrophic pyloric stenosis, patent ductus arteriosus, and hypospadias in male infants [37]. In adults, health outcomes as diverse as allergic wheeze [38], hypothyroidism [39, 40] and olfactory deficits [40] have all been linked to 2,4-D exposure.

In this analysis, several vulnerable population subgroups demonstrated an increased odds of higher urinary levels of 2,4-D associated with increased magnitude of 2,4-D use in agriculture. These subgroups include the youngest age group evaluated in NHANES surveys (children aged 6–11), as well as the oldest age group (adults aged 60+). Likewise, women of reproductive age also demonstrated increased odds of high exposure with rising agricultural use of 2,4-D.

Children may be at risk of higher exposure levels due to children’s play behaviors that include more time outdoors and on the floor, where there are higher quantities of dirt or dust particles with herbicide residue [41–43]. NHANES did not evaluate children under the age of 6 during the survey cycles included in this study. While the results here indicate a trend toward increasing exposure as age decreases, the data may not generalize to children under age 6 because of differences in types and amounts of food eaten, the ratio between amount of food eaten and unit of body weight, and the amount of accidental herbicide ingestion through play and mouthing behaviors [24]. This gap in knowledge is of critical concern because current knowledge of 2,4-D exposure dynamics [15] suggest that these differences may place younger children at even greater risk for higher exposure than their older counterparts. Further, young children are generally at higher risk for adverse health and developmental outcomes due to physiologic and developmental differences from adults [24]. Biomonitoring studies of 2,4-D exposure in pregnant women and young children are needed because of their unique vulnerabilities to both exposure and adverse health outcomes.

Only a small number of participants classified as agricultural workers were included in this study (*n* = 139). As expected, they were significantly more likely to have higher urinary 2,4-D concentrations compared to participants not classified as agricultural workers. This correlates with previous research on exposure to 2,4-D and other pesticides in agricultural workers [44, 45].

Non-Hispanic White participants had increased odds of higher exposure in both unadjusted and adjusted models compared to participants of other races/ethnicities. Because the models controlled for other variables related to socioeconomic status (SES), the differences probably cannot be explained entirely by factors related to SES. Although Non-Hispanic White participants had the highest odds of exposure compared to all other racial/ethnic groups, the difference was greatest between Non-Hispanic White and Non-Hispanic Black participants. It is possible that racial differences in geographic distribution, in particular legacy effects of racism resulting from formal and informal real estate redlining and segregation [46–48] may play a role in the differences in exposure between Black and White survey participants.

Race was included in this analysis recognizing it may serve as a proxy variable for geographic location and proximity to agricultural land and non-agricultural managed greenspace, including residential lawns [49–51]. In 2003, 2,4-D was applied at higher rates to American lawns and greenspaces than any other household herbicide on market [52]. The increase of southern Black migrants into northern cities through the 1960’s and 70’s drove many White families into suburban areas, where each home was often accompanied by a lawn in the front or back, and sometimes both [53]. Even in cities, urban greenspace is likely to be disproportionately distributed to White residents [54]. While these forms of structural racism often explain higher environmental exposure burdens among Black Americans [55–57], in this case, because non-agricultural uses relate primarily to lawns and green space, they may explain the apparent lower exposure burden among Black and other minority NHANES participants compared to Whites.

There was not a significant difference in biomarker levels by time of year of testing. It is expected that exposures are likely higher during the spray season for some people because of increased inhalation and dermal exposures due to proximity to land in agricultural use. However, NHANES reports testing season data in 6-month intervals that do not directly align with herbicide spraying season, which may explain why differences by time of year of testing were not statistically significant. The lack of control for geographic location in the analysis may have attenuated actual differences, or exposure may occur primarily through sources not dependent on proximity to agricultural land, such as through dietary sources or nearby lawn care applications.

There are some important limitations of this study, including that it did not evaluate health endpoints. The purpose of this study focused on determining whether changes in agricultural uses of 2,4-D are affecting human exposures. Variable limits of detection of 2,4-D biomarkers across survey cycles and a high percentage of biomarker values below the LOD affect the quantification of population exposure; it was not possible to evaluate associations with the mean population exposure level or its changes over time. Because 2,4-D has an expected half-life in humans of 10.2–28.5 h and is nearly completely cleared within 3 days [1], one-time spot urine samples provide information on a window of exposure immediately prior to sample collection but reflect neither the expected high variability in exposure levels from individual to individual, nor seasonal changes.

With these recognized limitations, studies tracking associations between agricultural pesticide application and human pesticide exposures are scant. As far as we are aware, this is the first study to evaluate 2,4-D biomonitoring levels and agricultural use of 2,4-D in a large nationally representative survey. In 2020, agricultural use of 2,4-D reached 33.3 million pounds nationally, reflecting a nearly 200% increase over the 2002 level (see Additional file 1). This rate of growth in the last two decades, however, will likely be dwarfed by the rate of growth and absolute annual increases in total pounds of 2,4-D applied in the next decade. Particularly sharp increases are expected in the next 3–5 years on soybean and cotton crop acres as the supply of 2,4-D-tolerant DuoEnlist™ seeds expands.

2,4-D serves as a sentinel for anticipated changes in other herbicide exposure levels, the application of which are changing in concert with changes in 2,4-D use (see Additional Files 5 and 6). Many of these herbicides have never been included in NHANES or other national biomonitoring studies. Continued monitoring of urinary 2,4-D levels by NHANES is strongly recommended, along with other herbicides that are increasing in use and potentially in exposure (e.g., glufosinate, dicamba, and 4-hydroxyphenylpyruvate dioxygenase (HPPD) inhibitors), as these will be used in progressively higher amounts over the coming years [58–60]. If these application trends unfold as predicted, the analyses reported here signal higher exposures to humans in the near future.

## Conclusion

Given substantial growth in agricultural use of 2,4-D since 2002 and the prospect of more significant growth through around 2030, the reported association between 2,4-D crop application and human biomonitoring levels is worrisome, particularly for vulnerable populations. Because herbicide use is rising, focused biomonitoring and epidemiological evaluation are needed to identify whether and how use and exposures are related to adverse health outcomes among vulnerable populations (particularly children and women of childbearing age) and highly exposed individuals (such as farmers, other herbicide applicators, and their families).

## Supplementary Information

**Additional file 1.** Pounds of 2,4-D Applied on National Acres, 1991–2014. A table of the pounds of 2,4-D applied over time by crop.

**Additional file 2.** Individual Analytes of 2,4-D as reported in NASS Chemical Use Surveys. A table of individual 2,4-D analytes as reported in NASS Chemical Use Surveys.

**Additional file 3.** Percent National Acres Treated with Glyphosate by Crop Over Time. A table of demonstrating the change in the percent of acres of selected crops treated with glyphosate over time.

**Additional file 4.** Percent National Acres Treated with Glyphosate. A figure showing the percent national cotton and soybean acres treated with glyphosate over time.

**Additional file 5.** Percent Soybean National Acres Treated with Top Herbicides Over Time. A figure showing the percent national soybean acres treated with top herbicides over time.

**Additional file 6.** Percent Corn National Acres Treated with Top Herbicides Over Time. A figure showing the percent national corn acres treated with top herbicides over time.

**Additional file 7.** Percent Crop Acres Treated with 2,4-D. A figure showing the percent national crop acres treated with 2,4-D over time.

**Additional file 8.** Pounds of 2,4-D Applied. A figure showing the pounds of 2,4-D applied over time for major crops.

## Data Availability

The datasets generated and/or analyzed during the current study are available in the NHANES repository, https://www.cdc.gov/nchs/nhanes/index.htm, and the Pesticide Use Data System repository, https://hygeia-analytics.com/pesticides/usage/puds-the-pesticide-use-data-system/.

## Abbreviations

2,4-D: 2,4-Dichlorophenoxyacetic acid
AHS: Agricultural Health Study
CDC: U.S. Centers for Disease Control and Prevention
EPA: U.S. Environmental Protection Agency
LOD: Limits of Detection
NASS: National Agricultural Statistics Service
NHANES: National Health and Nutrition Examination Survey
PUDS: Pesticide Use Data System
SES: Socioeconomic status
USDA: United States Department of Agriculture
