# Supplementary material for: Association between increasing agricultural use of 2,4-D and population biomarkers of exposure: findings from the National Health and Nutrition Examination Survey, 2001–2014
Source: Environ Health. 2022 Feb 10;21:23. doi: 10.1186/s12940-021-00815-x (PMC8830015; doi:10.1186/s12940-021-00815-x)
Supplement: Supplementary file 2 — Additional file 2. Individual Analytes of 2,4-D as reported in NASS Chemical Use Surveys. A table of individual 2,4-D analytes as reported in NASS Chemical Use Surveys. [file 12940_2021_815_MOESM2_ESM.docx]

| **Additional file 2. Individual Analytes of 2,4-D as reported in NASS Chemical Use Surveys** | |
| --- | --- |
| PC Code | Analyte |
| 030001 | 2,4-D |
| 030016 | 2,4-D, Diethamine salt |
| 030019 | 2,4-D, Dimethamine salt |
| 030025 | 2,4-D, Isopropanolamine salt |
| 030035 | 2,4-D, triisopropanolamine Salt |
| 030053 | 2,4-D, Butoxyethanol ester |
| 030063 | 2,4-D, 2-ehe |
| 030066 | 2,4-D, Isoprop ester |
| 030801 | 2,4-DB |
| 030819 | 2,4-DB, Dimethamine salt |
| 031419 | 2,4-DP, Dimethamine salt |
| 051505 | 2,4-D, Choline salt |
| 030034 | 2,4-d, triethylamine salt |
| Source: USDA NASS Quickstats Database | |
