# Supplementary material for: Association between increasing agricultural use of 2,4-D and population biomarkers of exposure: findings from the National Health and Nutrition Examination Survey, 2001–2014
Source: Environ Health. 2022 Feb 10;21:23. doi: 10.1186/s12940-021-00815-x (PMC8830015; doi:10.1186/s12940-021-00815-x)
Supplement: Supplementary file 3 [file 12940_2021_815_MOESM4_ESM.docx]

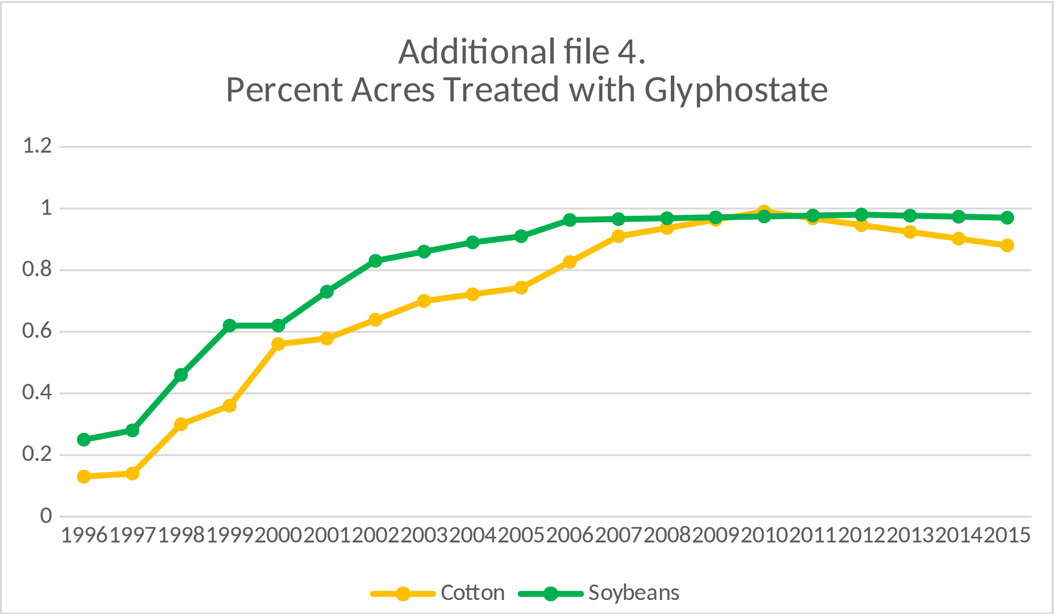
Additional file 4.
Percent Acres Treated with Glyphostate
1.2
1
0.8
0.6
0.4
0.2
0
1996 1997 1998 1999 2000 2001 2002 2003 2004 2005 2006 2007 2008 2009 2010 2011 2012 2013 2014 2015
Cotton
Soybeans
