# Supplementary material for: Association between increasing agricultural use of 2,4-D and population biomarkers of exposure: findings from the National Health and Nutrition Examination Survey, 2001–2014
Source: Environ Health. 2022 Feb 10;21:23. doi: 10.1186/s12940-021-00815-x (PMC8830015; doi:10.1186/s12940-021-00815-x)
Supplement: Supplementary file 5 [file 12940_2021_815_MOESM6_ESM.docx]

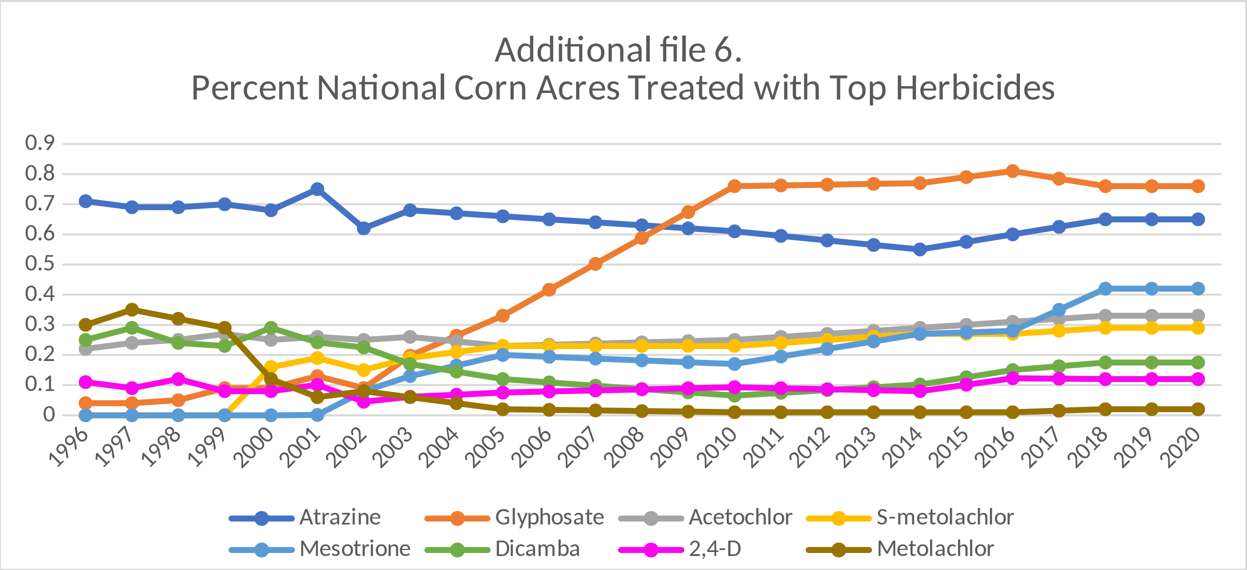
Additional file 6.
Percent National Corn Acres Treated with Top Herbicides
0.9
0.8
0.7
0.6
0.5
0.4
0.3
0.2
0.1
0
1996
1997
1998
1999
2000
2001
2002
2003
2004
2005
2006
2007
2008
2009
2010
2011
2012
2013
2014
2015
2016
2017
2018
2019
2020
Atrazine
Glyphosate
Acetochlor
S-metolachlor
Mesotrione
Dicamba
2,4-D
Metolachlor
