# Supplementary material for: Association between increasing agricultural use of 2,4-D and population biomarkers of exposure: findings from the National Health and Nutrition Examination Survey, 2001–2014
Source: Environ Health. 2022 Feb 10;21:23. doi: 10.1186/s12940-021-00815-x (PMC8830015; doi:10.1186/s12940-021-00815-x)
Supplement: Supplementary file 7 [file 12940_2021_815_MOESM8_ESM.docx]

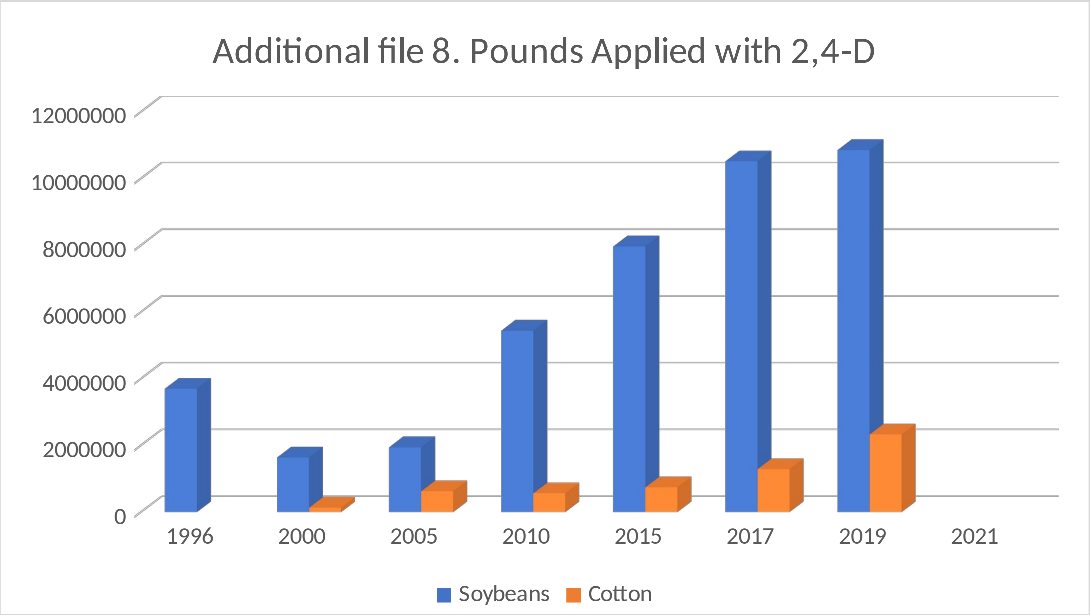
Additional file 8. Pounds Applied with 2,4-D
12000000
10000000
8000000
6000000
4000000
2000000
0
1996
2000
2005
2010
2015
2017
2019
2021
Soybeans
Cotton
